# The Dimensions of Organizational Character and Its Impacts on Organizational Performance in Chinese Context

**DOI:** 10.3389/fpsyg.2018.01049

**Published:** 2018-06-22

**Authors:** Dengke Yu, Huan Xiao, Qiushi Bo

**Affiliations:** School of Management, Nanchang University, Nanchang, China

**Keywords:** organizational character, organizational performance, organizational management, multi-ordered logit regression model, Chinese context

## Abstract

As an organic entity, organizations are similar to humans, having unique organizational character which constitutes a source of competitive differentiation. This study aimed to explore the dimensions of organizational character and measure its impacts on organizational performance in a Chinese context. Since several previous studies have developed the definition and constitutions of organizational character in the context of developed countries such as America and England, this indigenous study provides new evidence from the perspective of an emerging economy. A research model using a qualitative analysis method was proposed to define the dimensions of organizational character. The connection between organizational character and organizational performance was empirically tested by a multi-ordered logit regression analysis with a survey of 205 observations in Chinese enterprises. The dimension of organizational character was finally extracted and summarized as six aspects including enterprise, conscientiousness, innovation, agreeableness, democracy, and Boy Scout. The results of the empirical analysis showed that the formation and cultivation of organizational character would directly improve an organizations' business performance as well as their growth potential. It is worth noting that a special part of organizational character, which may depend more on national culture or institutional background than organizational individuals, also has an impact on organizational performance. The findings can provide practical implications for Chinese companies and multinational companies that do or plan to do business in China. Entrepreneurs are suggested to make effective decisions on the cultivation of organizational character, since different types and specific levels of organizational character may have significantly different effects on organizational performance. This paper explored a novel theory to explain the antecedents of organizational performance, and could inspire scholars to expand the sources of organizational competitive advantage in the future.

## Introduction

Think about the following slogans of TV advertisements delivered by some famous Chinese companies over the past few years:

Gone in a different way (Metersbonwe)
Make the change (LiNing)
Haier, made in China (Haier)
Born for the fancier (XIAOMI)

There are also innumerable similar advertising slogans not listed here. The public is impressed with these slogans because people can perceive the distinct personality of the organizations as well as their products and brands between the lines. However, only using these deliberate statements is not enough to be deeply rooted in the mind, it also requires products and services, staff, organizational image and so on to be consistent with the slogans so that people would then see them as a whole, which shows the distinctive personality traits of a company. In other words, as an organic entity, every company not only consists of assets such as staff, technology, and data, but also has a unique organizational character just like a human being (Machen, 1911). The relatively stable organizational characteristics that each company forms in the course of development would fundamentally distinguish it from other organizations (Chun and Davies, 2006). Thus, it is worth deeply exploring the mechanisms of the formation and operation of organizational character.

In fact, along with increasing product homogeneity in many industries, companies are usually faced with the dilemma of relying solely on providing high-quality products or services that are also difficult to assist them in maintaining sustainable competitiveness. At the same time, together with the adjustment of consumption structure, consumption patterns gradually shift from material consumption to spiritual consumption, and consumers pay more attention to the lifestyle, status, and characteristics of the corporation. Consumers are more willing to measure the characters demonstrated by the corporate world from their self-cognition so as to make better consumption decisions. Only companies that can better strengthen the self-cognition of consumers can better stimulate their consumption desires, improve product satisfaction, and even enhance their loyalty. It requires companies to be clear about what they have to do and what characteristics and personalities should be displayed so as to leverage organizational characters to continuously create profits.

This can also be a good explanation for the common phenomena nowadays that some incumbent companies with rich resources, excellent technology, and strong strength have suddenly failed in the competition. Instead, some new companies such as Didi and XIAOMI are occupying the markets at an unforeseen speed that is not commensurate with their historical accumulation. Organizational character, a common personality of different individuals in an organization that can be recognized by the outside world, has become one of the key differences between different organizations and has developed into an important source of differentiated competitive advantage (Resnick, 2003). As proposed by previous studies, organizational character is a relatively broad concept involving the diversity of corporate strategies, the uniqueness of organizational culture, the specificity of corporate products, and the development of employees' personalities (Moore, 2005). Due to these particularities, the differentiated competitive strategy led by organizational character has become an important way for some companies to break the existing market equilibrium and create opportunities for the sustainable development of organizations.

As an independent theoretical concept, organizational character's theoretical legitimacy and practical value depends largely on whether it can provide a unique contribution that is different from the existing theories. As far as the theoretical connotation is concerned, organizational character and organizational culture have a certain connection, but also have significant differences (Coutinho and Moraes, 2015). It is often manifested that individuals would be affected by the surrounding organizational culture when they enter an organization. Further, an obvious conclusion can be drawn that organizational culture makes significant effects on organizational character in a socialization process (Shiyi et al., 2008). However, researchers have repeatedly emphasized their essential differences. Organizational culture focuses on explaining the internal social and psychological environment of an organization from the perspectives of values and behaviors, but it is not embedded in a human being in essence. It is often alienated from specific individuals through providing a force to lead, restrict, or regulate individual behaviors. Conversely, the concept of organizational character not only includes an organization's internal personality and its formation mechanism, but also concerns the perception of employees and external stakeholders on organizational personality. It emphasizes that the intrinsic or perceived organizational characters are all relatively stable and regular in a period of time. Humanistic organizational character is always embedded or internalized into individual personality, but cannot exist independently (Denison, 1996). The differences and connection of organizational culture and organizational character are shown in Table 1.

**Table 1:** Differences and connection of organizational culture and organizational character.

**Concept**	**Theoretical root**	**Content**	**Perpetual object**	**Connection**
Organizational culture	Sociology and anthropology	Social and psychological environment	Entrepreneurs and employees	Organizational culture shapes organizational character and the latter would also affect the former in a continuous interaction
Organizational character	Psychology	Internal psychological characteristics and external perception	Entrepreneurs and employees as well as customers, the public and other stakeholders	

Although the concept of organizational character has been proposed by Western scholars in several previous studies (Birnholtz et al., 2007; Wen and Xie, 2009; Bo et al., 2017), the research effort on this issue is relatively scarce, especially in developing countries. Moreover, few scholars have studied the antecedent factors of organizational performance from the perspective of organizational character. In China, such studies have not been published up to now according to our document retrieval. There are only a few related studies that directly quote the scale of organizational character dimensions of developed countries. These studies ignored the consistency and differences of social culture and market institutions, and so their findings are controversial and further studies are needed for confirmation. Consequently, we tried to conduct an indigenous study in the Chinese context to fill those gaps and develop the organizational character theory.

Studying Chinese enterprises is especially significant for some reasons. First, China, as a representative of the Confucian culture, has obvious differences from Western countries such as the United States, which advocates the culture of “individualism.” Second, the intensive intervention of Chinese governments on the market can inevitably leave traces on the formation of organizational characters. Finally, China's economic development stage, i.e., new economy era, determines the diversification of Chinese companies business environment. Furthermore, the integration of Chinese and Western culture makes the organizational character of Chinese enterprises complex, diversified, and interesting. Therefore, the indigenous study of organizational character in a Chinese context will provide rich theoretical and practical implications.

Hence, we aimed to address the following two research questions in this paper:

RQ1: What are the dimensions of organizational character in a Chinese context?
RQ2: What is the relationship between organizational character and organizational performance in a Chinese context?

Therefore, we summarized the definition, dimensions, and effects of organizational character through a content analysis based on abundant research literature about organizational character, and proposed a new conceptual model and made an empirical analysis for measuring the impacts of organizational character on organizational performance. In this way, this paper can help organizations cultivate suitable organizational characters to realize their differential strategies to enhance their sustainable competitive advantage and support their sustainable development. The remainder of the paper is organized as follows. Section Literature Review presents the literature review. Section Methods deals with the methodology employed to collect and analyze the survey data. Results of the analysis are presented in Section Modeling and Results, followed by some discussions. Finally, the theoretical and practical implications of the findings as well as the future research directions are mentioned in the last section.

## Literature review

For an individual, the closest personality trait with social relationship is their character (Fu and Ning, 2014). It is generally believed that human behavior is consistent with and predictable by analyzing one's character, which is an important aspect of personality referring to individual psychological characteristics as well as the mechanisms of the collection with organizational and relative durability (Larsen and Buss, 2007). The concept of organizational character has existed for a long time though scholars have differed in their understandings of it due to different social and culture backgrounds. Shee and Abratt (1986) proposed early on that organizational character could be viewed as the sum of organizational behaviors and intellectual characteristics. Slaughter et al. (2004) defined organizational character as the personality trait of organizations, which was similar to individual personality, that can be perceived by the outside world. However, Otto et al. (2006) argued that, unlike individual personality, organizational character should reflect the expectations of customers, suppliers, shareholders, and employees regarding organizational performance. Similarly, Slaughter et al. (2004) also suggested that there was a certain difference between organizational character and individual personality. Specifically, some existing research have confirmed that almost every survey tool of individual personality is not applicable to the measurement of organizational character. However, most scholars agree that organizational character contains both moral and social aspects (Wright, 2008). Hence, the concept can be considered as a multidimensional construct.

In terms of component analysis, some researchers have identified and categorized the dimensions of organizational character. One of the earliest empirical studies by Spector (1961) identified six dimensions of organizational personality: dynamic, cooperative, business-wise, successful, character, and withdrawn (Spector, 1961). Bridges (2000) drew on the knowledge of personality psychology and applied the theory of MBTI (Myers-Briggs Type Indicator) to the organizational level to make a more systematic study (Bridges, 2000). He categorized the details of organizational character into eight dimensions, namely, extraversion/introversion, sensing/intuition, thinking/feeling, and judging/perceiving. Those early measures for organizational character are rooted in actual operations and often directly use personality vocabulary in individual psychology research. Thus, there are shortcomings in the theoretical, systemic, and scientific aspects. Recently, scholars have begun to study organizational character issues with a more scientific vision, e.g., developing organizational character dimensions in the true sense of the concept based on distinguishing differences from and the relationship with individual personality. In those studies, methodological tools can be divided into the deductive method and induction method. For example, through a deductive exploration, Fernández and Hogan (2003) pointed out that defining the distinct character of a great organization needed four value clusters: achievement type, safekeeping type, collaborative type, and creative type (Fernández and Hogan, 2003). Likewise, Moore (2015) proposed that organizational character was a relatively broad concept, which should include organizational strategy, organizational culture, product positioning, employee personality, and many other choice preferences (Moore, 2015). The deductive method is always based on personality type theory, while the induction method is often grounded in personality trait theory. For instance, Davies et al. (2004) selected 2,061 employees and 2,565 customers as a sample set for factor analysis, through which they summarized seven dimensions of organizational character: agreeableness, enterprise, competence, chic, ruthlessness, informality, and machismo (Davies et al., 2004). At the same time, Slaughter et al. (2004) completed an exploratory factor analysis based on 255 selected terms associated with organization personality, but she only extracted five dimensions including Boy Scout, innovativeness, dominance, thrift, and style. British scholar Otto et al. (2006) conducted an online survey of 64 corporations to identify the dimensions of organizational character, in that study, he named them as honesty, prestige, innovation, and power. To sum up, from the perspective of organizational governance, we know that some scholars have respectively studied the dimensions of organizational character in the context of different cultures, and in fact, the quantity and quality of dimensions are quite different, though a fraction of them are consistent in connotation. This implies that consistency and differences do exist among organizational character dimensions in different cultures, and indigenous research is necessary for comparative study and policy design.

The theorists of personality psychology have examined the consequences of individual personality at three levels: individual, interpersonal, and organizational (Ozer and Benetmartínez, 2006). Along this line, discussions on the organizational character's effect have also been conducted at three levels. At the individual level, Turban and Keon (1993) found that different organizational traits lead to different attractiveness for job applicants as people tend to choose companies that fit with their own personality (Turban and Keon, 1993). At the interpersonal level and on the basis of interactive psychology theory, Halfhill et al. (2008) argued that the impacts of organizational character on interpersonal relationships involved both positive and negative effects (Halfhill et al., 2008). In the early stage, organizational character would promote different employees to become more similar in personality traits, thus promoting coordination, communication, and cooperation among employees, and also reducing conflicts. However, the influence of organizational character on interpersonal relationships is not a simple linear trend. Along with the continuous development of organizations, the effects may also change; a high degree of homogeneity in character may increase interpersonal contradiction. At the organizational level, existing research has mainly focused on the organizational character in terms of organizational behavior, organizational reputation, and organizational image. Back in the 1950s, Newman proposed that organizational traits could explain and predict organizational behavior (Newman, 1953). Bridges (2000) showed that organizational members and customers would assess organizational reputation through the correct cognition of organizational character (Bridges, 2000). Van den Bosch et al. (2006) found that organizational characters strongly affected the way corporate visual identity was managed (Van den Bosch et al., 2006). The study of Love and Kraatz (2009) on the relationship between organization personality and organizational reputation further corroborated this view. The findings of Farrukh et al. (2007) showed that extroversion, agreeableness, and conscientiousness had positive impacts on affective commitment, while neuroticism and openness had negative impacts. In addition, several studies remind us that although researchers generally have a positive attitude toward organizational character, some scholars have also verified its negative consequences. For example, Miller (1991) suggested that a high degree of homogeneity in character may reduce organizational flexibility and cause organizations to fail in its adaptation to environmental changes (Miller, 1991).

In summary, it can be found that organizational character may determine organizational performance in many ways. However, the empirical research on this issue is fragmented and even fewer researchers have focused on the impacting processes and mechanisms. Moreover, like individual personality, the formation of organizational character may be influenced by the cultural context. The dimensions of organizational character as well as their interpretation would differ in different cultural backgrounds. However, existing studies on organizational character have mainly been conducted against a Western cultural background such as Britain and America. Since cultural differences between the East and West do exist, previous research results have not been able to be transplanted directly to Eastern countries such as China. Thus, this paper used an open questionnaire to explore the organizational character dimensions in the Chinese context, and examined the impacts of organizational character on organizational performance through an empirical study. This paper hereby put forward a hypothesis: organizational character has a significant positive impact on organizational performance.

## Methods

Qualitative and quantitative research methods were used in this study. In the following two parts of data analysis, qualitative research including content analysis and coding study was first undertaken to summarize the dimensions of organizational character on the basis of data collection through an open questionnaire investigation, and second, we conducted an empirical analysis by using a multi-ordered logit regression model for measuring the impacts of organizational character on organizational performance.

### Measures

The “State-owned Capital Performance Evaluation Rules” promulgated by China in 1996 stipulates that corporate performance refers to the financial performance and operating performance, which is associated with a company's operating activities. Concretely, organizational performance is suggested to be measured by profitability and growth. In this study, to measure and compare the impacts of each organizational character dimension on organizational performance, we referred to the above rule to construct organizational performance and learnt from Andersen and Skjoett-Larsen (2009) and Jiangtao and Yabiao (2014) how to design the questionnaire items. Therefore, business performance and growth potential were applied to the measurement of organizational performance and set as the dependent variables in the following empirical model.

Business performance is defined here as the short-term financial performance of an organization during a certain period of operation. It reflects the current operating condition of the organization. Men and Tsai (2015) believed that public participation and intimate and easy-going corporate characteristics help to enhance organizational public relations, thereby increasing business performance.

Growth potential is defined here as the long-term performance that reflects the development potential of an organization. It represents the sustainable development trend of the organization as well as the degree of public recognition and expectation of this trend. Church et al. (2015) explored the relationship between personality characteristics and company development. He reminded and warned us that the character's role in organizational development was often ignored or underestimated.

Thus, two items were correspondingly used to measure organizational performance. These were “the current financial performance of my organization is very high compared with other organizations in the same industry” and “my organization has great potential for development.” We used a 5-point Likert-type scale (ranging from 1 = strongly disagree to 5 = strongly agree) to rate the items.

The approach for measuring organizational character relies on the metaphor of treating organizations as human beings. Since Aaker (1997) developed a brand personality scale, similar analogism has been generally used in academia (Aaker, 1997). “Supposed a brand is a person, what personality do you think he or she has?” Such questions do not have much difficulty for respondents to associate and analogize (Aaker, 1997). We therefore used this brand personality scale for reference. Each respondent was required to imagine that the organization in which they worked “has come to life as a human being” and to fill five adjectives or phrases in their first thoughts that could best reflect the organization's specific trait.

### Samples

In this study, a total of 250 questionnaires were randomly sent out through the “Star Questionnaire” network, which is a specialized online service institution for questionnaire surveys in China. During the 12 years of its operation, more than 1.5 billion surveys have been completed with its professional service. It can distribute and collect a large number of high quality questionnaires in a relatively short time through a variety of channels such as streaming media, web, and mail. Respondents are often discovered through a unique cooperative recommendation model. Currently, its users are close to 24 million, and many Chinese scholars have completed their survey studies with the help of this institution.

Fortunately, 239 copies were collected in a week. After eliminating the invalid questionnaires where excessive entries were omitted or the respondent's attitude was not good, e.g., selecting the same option for all questions, we finally obtained 205 valid questionnaires with the effective response rate of 86%. According to the basic information filling in the questionnaires, 43% of the respondents were male, and 57% were female; 24% of the respondents worked in their current workplace for 1–3 years and 73% for more than 3 years; 93% of the respondents had received a bachelor's degree or above. More than 70% of the respondents had a bachelor's degree and simultaneously had worked in the current organization for more than 3 years. This ensured that the respondents had enough perception and mature understanding of the organizations in which they worked. In addition, 27% of the respondents were from state-owned companies and the others were from private companies; 44% of the respondents were engaged in management, 32% worked in the R&D department, 10% were in the production department, and the other 14% worked in the marketing department; 45% were from high-tech companies, and the others are not; 20% were from large companies, 65% from medium-sized companies, and the remaining 15% were from small companies; finally respondents were distributed in 21 provincial areas in China according to the sample statistics.

### Analytical stages

To identify the dimensions of organizational character and measure their impacts on organizational performance, we designed the study process in the following two stages:

Stage 1: Open questionnaire survey and content analysis method were used to conduct qualitative analysis of organizational character dimensions in the Chinese context.
Stage 2: Multi-ordered logit regression method was used to analyze the impacts of organizational character on organizational performance.

## Modeling and results

We first classified the terms of organizational character. Based on the statistical analysis of term frequency and coding analysis of the term's content, six dimensions of organizational character were obtained. Furthermore, a multi-ordered logit regression model was constructed to measure the effects of each dimension on organizational business performance and growth potential, respectively. In this model, organizational scale and organizational ownership were employed as control variables. In this study, the organizational scale covered three degrees, i.e., large enterprises, medium-sized enterprises, and small and micro enterprises, which were identified in terms of employee numbers. The organizational ownership was considered from two aspects, that is, state-owned enterprises and private enterprises.

### Dimensional recognition

Human personality or brand personality in previous studies has been generally divided into five or more dimensions. Similarly, we expected organizational character to be a multidimensional construct. Hinkin (1995) suggested that an inductive approach should start with qualitative interviews and use content analysis of relevant sources to identify themes and core categories (Hinkin, 1995). Referring to the above literature, the inductive approach was used to generate dimensions on the basis of analyzing descriptive terms in this study.

In order to increase the repeatability of the research procedure, the vocabulary encoding part was carried out by three authors at the same time. After the coding was completed, we measured the level of coding consistency, which was up to 90%. This indicated that the encoding analysis was of high reliability. Finally, the coding results of the three authors were comparatively reviewed, selected, cleaned, and combined to form an end result.

Concretely, data were processed in the following steps:

1. A total of 205 questionnaires in each of which five words or phrases were filled to describe organizational character were recovered. A total of 1,025 entries were collected.
2. We looked through these entries preliminarily and replaced the obvious synonyms with the corresponding core terms according to the codebook in Table 2. For example, entries such as conscientiousness, responsibility, pious, duteous, elaborateness, and responsible, etc. were all replaced by “conscientiousness”; and entries such as harmony, harmonious, united, peace, concord, and amicable, etc. were all replaced by “harmony.”
3. We analyzed the core terms and entries by their frequency statistics, and the entries which appeared only once were cleaned in order to reduce chanciness and facilitate the next step of encoding work. After filtering, a total of 775 entries and a total of 52 core terms were left. All core terms were sorted in descending order of frequency, 18 core terms with a frequency of more than 10 were each taken as a category, as shown in Table 3.
4. Drawing lessons from previous literature and the conceptual model, the selected core terms were classified and coded into categories on the basis of content analysis. Research processes of frequency statistics, combination, and induction were carried out and finally six new constructs were obtained (see Table 4). These constructs were seen as the dimensions of organizational character. Therefore, the coded dimensions of organizational character were enterprise, conscientiousness, innovation, agreeableness, democracy, and boy scout. The new constructs which were named by nouns, rather than adjectives, were built to capture the underlying meanings of the dimensions of organizational character. The constructs were checked with a small sample of return-visited respondents to identify if there was any improvement or change to the labeling process. Results proved the dimensional theory saturated.

**Table 2:** Codebook for extracting core terms.

**Criteria**	**Core terms**	**Synonyms**	**Antonyms**
1. The adjective or noun of a person's personality 2. Refer to the “modern Chinese dictionary” and the “Collins dictionary” 3. Increase some demographic adjectives 4. Increase the vocabularies of organizational image in previous related studies	Agreeable	Agreeableness, pleasant, delightful, jolly, comfortable	Unsuitable, uncomfortable, unpleasant
	Conscientious	Serious, conscientiousness, earnest, careful, cautious, meticulous	Indiscreet, rash, imprudent, impetuous, careless
	Cooperative	Coadjutant, synergetic, collaborative, cooperation, boy scout	Disoperative, uncooperative
	Democracy	Democratic, equal	Undemocratic, bureaucratic
	Developmental	Evolutive, promising, hopeful, aussichtsreich, prospective	Stagnant, moribund,
	Enterprise	Enthusiastic, aggressive, upward, active, energetic, proactive	Inactive, conservative
	Efficient	High-efficiency, businesslike	Inefficient, ineffective
	Harmony	Harmonious, concordant, amicable	Discordant, disharmonious
	Innovative	Innovation, creativity, original	Low-tech
	Integrity	Upright, righteous, truthful, guileless	Foxy, cunning, tricky, crafty,
	Just	Impartial, righteous, fair, candid	Unequal, unjust, partial
	Open	On-limits, exoteric, open-ended, enlightened	Conservative, fogyish, old-fashioned
	Reliable	Dependable, credible, faithful, responsible	Unreliable, trustless, irresponsible
	Unity	United, cohesive, unitive, solidarity	Ununited, disunity

**Table 3:** High frequency core terms.

**Core terms**	**Frequency**	**Core terms**	**Frequency**	**Core terms**	**Frequency**
Innovation	79	High-tech	24	Just	11
Harmony	58	Conscientiousness	22	Potential	11
Unity	43	Open	21	Youthful	11
Enterprise	30	Developmental	16	Cooperative	10
Stability	27	Democracy	16	Sociable	10
Efficient	25	Struggling	14	Integrity	10

**Table 4:** Coding processes for exploring organizational character dimensions.

**Dimensions (categories)**	**Core terms**	**Frequency**
Enterprise	Enterprise, upward, efficient Developmental, struggling, aggressive Youthful, energetic, vital, extrovert	186
Conscientiousness	Conscientiousness, normative, responsible, preciseness Stability, pragmatic, dedicated, integrity	135
Innovation	Innovation, creativity, R&D, High-tech, original Freedom, interesting, unique, fresh	128
Agreeableness	Warmth, friendly, sincere, agreeable, humanized Sociable, empathy, harmonious, concerned, pleasant	125
Democracy	Democracy, equality, just, fair Open, enlightened, comprehensive	103
Boy scout	Boy scout, cooperative, helpful, win-win, unity	98

To observe and analyze the six dimensions, it was found that there was a certain correlation between organizational character and individual personality. For example, the sense of conscientiousness was exactly consistent with the “conscientiousness” dimension of the “Big Five personality,” and agreeableness was very close to the “agreeable” dimension. More to the point, some dimensions in this paper were similar to those in previous studies, which are referred to in Table 5. It is worth pointing out that “democracy” seems to be a contextual dimension full of Chinese cultural characteristics and we have not found similar items in previous literature.

**Table 5:** The comparison of organizational character dimensions in this study and previous studies.

**Dimensions**	**Previous literature**
Enterprise	Davies et al. (2004), UK, Enterprise
Conscientiousness	Otto et al. (2006), UK, Honesty The “Big Five personality,” Conscientiousness
Innovation	Otto et al. (2006), UK, Innovation Slaughter et al. (2004), US, Innovation
Agreeableness	Davies et al. (2004), UK, Agreeableness The “Big Five personality,” Agreeable
Boy scout	Spector (1961), US, Cooperative Slaughter et al. (2004), US, Boy Scout

**Figure 1:**
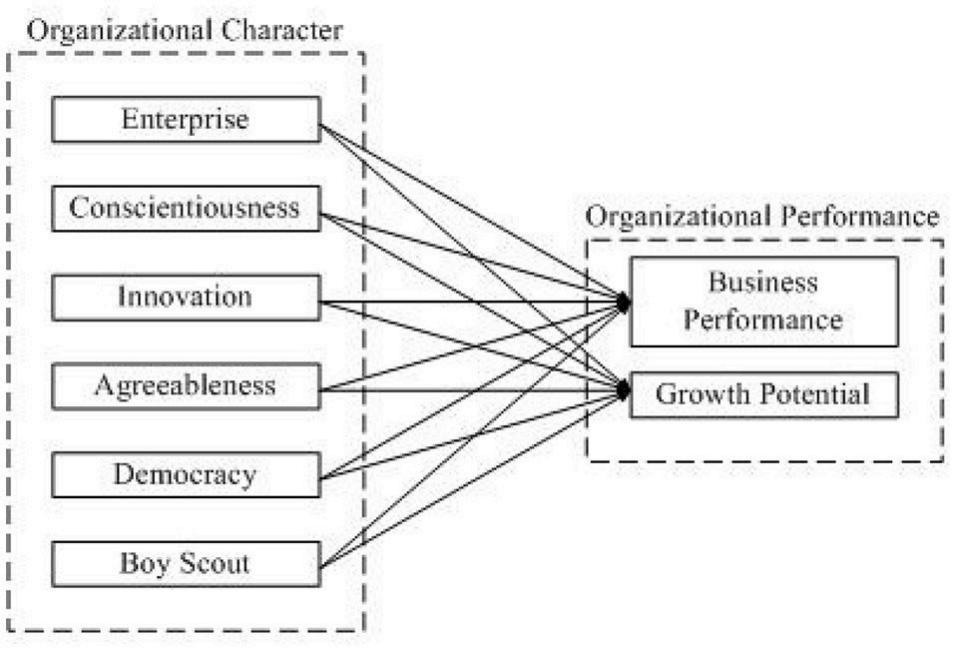
Conceptual model.

Finally, the authors constructed a conceptual model, as shown in Figure 1. This model allowed all five dimensions of organizational character to correlate with two outcome variables: business performance and growth potential. Using it as the baseline model, we aimed to verify the validity of the above theoretical constructs and measured the significant effects between each organizational character dimension and business performance as well as growth potential.

### Data processing

After coding the six dimensions of organizational character, the authors carried out the data processing again for the following empirical analysis. Inspired by Young (1981) and Sayago (2015), the specific data processing steps were set as follows (Young, 1981; Sayago, 2015):

1. Generating data. The authors converted the qualitative data with the respondents' answers into quantized data. Specifically, according to the six dimensions of organizational character, if a respondent positively mentioned the entries associated with a dimension, then we assigned a value of 1 to this dimension; if the respondent mentioned the entries negatively, we assigned −1 to the corresponding dimension; if no mention of this dimension, the assignment was 0. Thus, we generated a matrix data of 6 × 205.
2. Setting weights. The authors supposed that the first entry of a respondent's subconscious answer should be the most prominent organizational character they observed, followed by the second entry, and the fifth entry at the least. Therefore, the weight of the first entry was 5, the weight of the second entry was 4, and so on, and the weight of the fifth entry was 1.
3. Assigning weights to the data, each dimension was measured with the maximum value of 15 (= 5 × 1 + 4 × 1 + 3 × 1 + 2 × 1 + 1 × 1) and minimum value of −15 (= 5 × −1 + 4 × −1 + 3 × −1 + 2 × −1 + 1 × −1). Thereout, the data of dimensional variables have been assigned entirely.

### Reliability and validity analysis

The scale of organizational performance that reflects both business performance and growth potential is a typical reflective scale. Unlike this construct, each one of the six dimensions of organizational character can be seen as a complete cause to explain an organization's character from its own dimensional perspective. Six dimensions, which are mutually independent, jointly determine the conceptual operational definition of the organizational character as a whole. Therefore, we considered the construct of organizational character as a formative model.

The traditional tools for testing reliability and validity are not applicable to a formative model. In a formative model, it is always assumed that each of measurement variables has no error term, the correlations between measurement variables are exogenous and the correlation coefficients can be negative or zero. Therefore, the internal consistency between measurement variables is not suitable for a reliability test in a reflective model (Jarvis et al., 2003). Based on this consideration, the Cronbach's alpha value was not used to test the reliability of the organizational character construct. By referring to some relevant literature (Kishton and Widaman, 1994; Zhou et al., 2007), the “items parceling” method has been alternatively used to test its validity. To complete that, the original measurement variables of the organizational character were processed in a certain way as follows.

First, we undertook an exploratory factor analysis (EFA) of the organizational character, the results of which are shown in Table 6. Using the principal component analysis method, we proposed three factors and named them as OC1, OC2, and OC3, respectively. These factors represented three major virtual components of the organizational character. Then, according to the factor loading, items (which in this study are the dimensions of the organizational character) were extracted from each factor. The average value of the extracted items was calculated as the corresponding item parcel.

**Table 6:** Item parcels in EFA.

**Items**	**Item parcels**		
	**OC1**	**OC2**	**OC3**
Agreeableness	0.68	−0.47	−0.14
Democracy	0.49	0.04	0.34
Boy scout	0.47	0.26	−0.12
Innovation	0.01	0.64	0.64
Enterprise	−0.20	0.50	−0.66
Conscientiousness	−0.63	−4.65	0.46

Second, a confirmatory factor analysis (CFA) model, which covers organizational character and organizational performance as latent variables was conducted to test the psychometric properties of the scales. The results, shown in Table 7, show that the data fit well. The critical ratios of all factors (CMIN/DF = 1.68 < 5, GFI = 0.98 > 0.90, NFI = 0.96 > 0.90, IFI = 0.96 > 0.90, RMSEA = 0.058 < 0.08, RMR = 0.059 < 0.08) were greater than the suggested values. The fact that most of the standardized coefficients of the measurement variables were more than 0.70 indicated that the intrinsic quality of the model was well tested and the level selected was suitable for the measurement index of the latent variables. The composite reliability (CR) of both the organizational character and organizational performance being greater than 0.60 indicated that the model had good reliability of model construction. The values of average variance extraction (AVE) were all greater than 0.50, which indicated that the convergence validity of the measurement model was good. In addition, the Cronbach's alpha of organizational performance was 0.76, above the recommended level of 0.70. All in all, the results met the rules of thumb in construct reliability (Bollen and Lennox, 1991; Bernstein and Nunnally, 1994; Hair et al., 2006).

**Table 7:** Reliability and validity test.

**Latent variable**	**Measurement variable**	**Standardized coefficient**	**Cronbach's alpha**	**CR**	**AVE**
Organizational character	OC1	0.82^**^	–	0.60	0.82
	OC2	0.61^**^			
	OC3	0.87^**^			
Organizational performance	Business performance	0.64^**^	0.76	0.73	0.57
	Growth potential	0.86^**^			

CMIN/DF = 1.68 < 5, GFI = 0.98 > 0.90, NFI = 0.96 > 0.90, IFI = 0.96 > 0.90, RMSEA = 0.058 < 0.08, RMR = 0.059 < 0.08;

** *P < 0.01*.

Finally, considering the measurement of organizational character was an open-ended questionnaire, test–retest reliability is a preferable reliability estimation method for a formative model (Edwards and Bagozzi, 2000). Thus, the independent samples test was used to reflect the alternate-form reliability of the data by randomly allocating the samples into two groups in advance. The results are shown in Table 8. It can be found that all variables except democracy obeyed the homogeneity assumption of variance since all were not significant at the 0.10 level in Levene's test; all variables were not significant at the 0.10 level in the two-tailed test of the *t*-test. Thus, we considered that there was no statistical difference between the two groups in each dimension. It also showed that the alternate-form reliability of the data was acceptable.

**Table 8:** Independent samples test for measuring alternate-form reliability.

**Variables**	**Groups**	**Mean**	**St. D**	**Levene's test**		***T*****-test**	
				***F***	**Sig**.	***T***	**Sig. (2-tailed)**
Enterprise	1	1.97	2.50	1.20	0.27	−0.70	0.48
	2	2.24	2.86				
Conscientiousness	1	0.85	1.89	0.22	0.64	−0.40	0.69
	2	0.96	1.98				
Innovation	1	2.17	3.62	0.13	0.72	−0.78	0.44
	2	2.54	3.16				
Agreeableness	1	1.88	2.88	1.76	0.19	0.69	0.49
	2	1.62	2.60				
Democracy	1	0.70	3.27	8.37	0.00	0.61	0.55
	2	0.47	1.87				
Boy scout	1	1.99	3.34	2.64	0.11	−0.16	0.87
	2	2.06	2.62				

### Descriptive analysis

Descriptive analysis (see Table 9) was used to determine the distribution homogeneity, correlation, and direction of variables.

**Table 9:** Descriptive statistics of variables.

**Variables**		**Mean**	**St. D**	**Correlation**								
				**2**	**3**	**4**	**5**	**6**	**7**	**8**	**9**	**10**
Dependent variables	1. Business performance	2.56	0.62	0.50^**^	0.26^**^	0.17^*^	0.20^**^	0.02	0.12	0.07	0.05	0.06
	2. Growth potential	2.59	0.68		0.26	0.15^**^	0.28^**^	0.13^*^	0.28^**^	0.10	−0.07	0.27^**^
Independent variables	3. Innovation	2.10	2.68			0.01	−0.04	−0.15^*^	0.06	−0.10	0.07	0.14^*^
	4. Boy Scout	0.90	1.93				−0.03	0.02	0.01	−0.13	−0.11	0.14^*^
	5. Enterprise	2.35	3.39					−0.14^*^	−0.03	−0.10	−0.03	0.22^**^
	6. Agreeableness	1.76	2.74						0.12	−0.16^*^	−0.09	0.04
	7. Democracy	0.60	2.67							−0.08	−0.02	0.12
	8. Conscientiousness	2.03	2.99								−0.08	−0.05
Control variables	9. Organizational size	1.75	0.47									0.40^**^
	10. Organizational ownership	1.95	0.58									1

** P < 0.01;

* *P < 0.05*.

The following two findings were drawn from the descriptive analysis:

1. From the correlation coefficient, the correlations between organizational character and business performance as well as growth potential were mostly significant, and there may be a certain correlation between organizational ownership and organizational character. In other words, organization ownership may be a valid control variable.
2. The correlations among the different dimensions of organizational character were not significant, which indicated that most of the dimensions were mutually independent. This proved again that the construct of organizational character is formative. Unlike the reflective model, the formative model does not emphasize that there must be significant correlation between its formative factors.

### Statistical analysis and results

The ordinary least squares (OLS) method is usually used to obtain the optimal linear unbiased estimation, but several assumptions are required. However, one of these assumptions, the variables being continuous, were not satisfied in this study. While the data of the dependent variable were discrete, the OLS method would generate a serious inference problem. In this case, maximum likelihood estimation techniques, e.g., logit or probit, are usually the preferred tools. Since the dependent variables in this study were ordered categorical variables, the authors chose to construct a multi-ordered logit model for statistical analysis.

The multi-ordered logit model is as follows:

(1) $$Y_{i}^{*}=\sum_{k=1}^{8}\beta_{k}X_{ik}+\varepsilon_{i},\;y_{i}=\{\begin{array}{l} 1\;if\;Y_{i}{}^{\text{*}}\leq C_{1}\\ 2\;if\;C_{1}<Y_{i}{}^{\text{*}}\leq C_{2}\\ 3\;if\;Y_{i}{}^{\text{*}}\geq C_{2} \end{array}$$

where $Y_{i}^{*}$ is an unobservable latent variable; *y*_*i*_ is the observation value; and ε_*i*_ is an independent and identically distributed random variable of which the distribution function obeys extreme value distribution. The *C*_1_, *C*_2_(0 ≤ *C*_1_ ≤ *C*_2_) and β_*k*_ are parameters that need to be estimated. According to the above model, the probability of each *y*_*i*_ can be expressed as:

(2) $$\begin{array}{l} \mathrm{Pr}\left(y_{i}=1\right)=\text{Pr}\left(\left({\text{Z}}_{i}\text{+}\varepsilon_{i}\right)\leq C_{1}\right)=\text{Pr}\left(\varepsilon_{i}\leq \left(C_{1}-{\text{Z}}_{i}\right)\right)\\ \mathrm{Pr}\left(y_{i}=2\right)=\text{Pr}\left(C_{1}\leq \left({\text{Z}}_{i}\text{+}\varepsilon_{i}\right)\leq C_{2}\right)=\text{Pr}\left(\left(C_{1}-{\text{Z}}_{i}\right)<\varepsilon_{i}\leq \left(C_{2}-{\text{Z}}_{i}\right)\right)\\ \mathrm{Pr}\left(y_{i}=3\right)=\text{Pr}\left(\left({\text{Z}}_{i}\text{+}\varepsilon_{i}\right)\geq C_{2}\right)=\text{Pr}\left(\varepsilon_{i}\geq \left(C_{2}-{\text{Z}}_{i}\right)\right) \end{array}$$

where $Z_{i}=\sum_{k=1}^{8}\beta_{k}X_{ik}$. By means of maximum likelihood estimation, the limit values of *y*_*i*_ and β_*k*_ are estimated simultaneously. The definitions of the variables in the model are shown in Table 10.

**Table 10:** Variable description of logit model.

**Variables**	**Explanations**	**Range of values**
Y_1_^*^	Business performance	1 = low; 2 = medium; 3 = high
Y_2_^*^	Growth potential	
X_1_	Enterprise	X_i_ = −15, −14, …0, 1, 2…15
X_2_	Conscientiousness	
X_3_	Innovation	
X_4_	Agreeableness	
X_5_	Democracy	
X_6_	Boy scout	
X_7_	Organizational size	1 = small; 2 = medium; 3 = large
X_8_	Organizational ownership	1 = state-owned company; 2 = private companies

According to the above multi-ordered logit regression model, the authors used SPSS 17.0 to fit the model by using the maximum likelihood estimation method, and then the effects of organizational character on organizational performance were measured. The results are shown in Table 11. According to the table, we know that the organizational differences in terms of organizational size and organizational ownership would not significantly increase the interpretation of the impacts of organizational character on organizational performance.

**Table 11:** The fitted coefficients of the model.

**Variables**		**Dependent variable (Business performance)**				**Dependent variable (Growth potential)**				**Dependent variable (Organizational performance)**			
		**Coef**.	**Std. Err**	**Wald**	**Sig**.	**Coef**.	**Std. Err**	**Wald**	**Sig**.	**Coef**.	**Std. Err**	**Wald**	**Sig**.
Independent variables	Enterprise	0.16^**^	0.05	10.48	0.00	0.23^**^	0.06	15.08	0.00	0.21^**^	0.05	17.90	0.00
	Conscientiousness	0.11^*^	0.05	4.90	0.03	0.17^**^	0.06	8.15	0.00	0.14^**^	0.05	7.78	0.01
	Innovation	0.27^**^	0.07	15.14	0.00	0.35^**^	0.09	17.10	0.00	0.35^**^	0.07	26.85	0.00
	Agreeableness	0.08	0.06	1.79	0.18	0.16^**^	0.06	6.34	0.01	0.13^*^	0.05	5.87	0.02
	Democracy	0.06	0.05	1.14	0.29	0.19^**^	0.07	7.60	0.01	0.12^*^	0.05	5.20	0.02
	Boy scout	0.27^**^	0.10	7.35	0.01	0.17	0.10	2.89	0.09	0.26^**^	0.09	10.39	0.00
Control variables	Size	0.38	0.29	1.74	0.19	0.17	0.32	0.30	0.58	0.28	0.26	1.16	0.28
	Ownership	−0.18	0.39	0.22	0.64	0.71	0.41	2.97	0.08	−0.06	0.32	0.03	0.86
^**^P < 0.01; ^*^P < 0.05		Number of obs = 205; LR chi^2^(8) = 39.37; Log likelihood = −584.62; Prob > chi^2^ = 0.00				Number of obs = 205; LR chi^2^(8) = 73.54; Log likelihood = −499.48; Prob > chi^2^ = 0.00				Number of obs = 205; LR chi^2^(8) = 69.53; Log likelihood = −424.26; Prob > chi^2^ = 0.01			

The variables concerning six dimensions of organizational character were used to measure their direct impacts on organizational performance. As shown in Table 10, except for “agreeableness” and “democracy,” other dimensions of organizational character had significant positive impacts on business performance. Among them, “innovation” (coefficient = 0.27, *p* = 0.00) and “boy scout” (coefficient = 0.27, *p* = 0.01) had relatively great impacts. Furthermore, all dimensions of organizational character had significantly positive effects on growth potential in organizations. The most obvious effects were from “innovation” (coefficient = 0.35, *p* = 0.00) and “enterprise” (coefficient = 0.23, *p* = 0.00). As a whole, all dimensions had significant positive impacts on organizational performance. Among them, “innovation” (coefficient = 0.35, *p* = 0.00) was not only a fundamental, but was also the most important component of organizational character from the perspective of raising performance. An interesting phenomenon was that “democracy” (coefficient = 0.12, *p* = 0.02), which is the dimension representing indigenous culture and contextual factor, played the weakest role in the impacting path.

The results indicated that the formation and cultivation of organizational character not only directly helped to improve organizational business performance, but also played an instrumental role in the enhancement of organizational growth potential. In general, organizational character provided a good explanation for organizational performance from both a short and long term perspective. Organizational character would be an essential factor for the formation and promotion of organizational performance. This finding is novel in theory and valuable in practice.

## Discussion

Compared with the fruitful achievements made in the research field of personality psychology to explain and predict individual behaviors, the concept of character at an organizational level has not been paid sufficient attention in the research fields of management. However, similar to personality psychology, organizations in a dynamic market competition environment may also face the rule of “organizational character shapes destiny.” Organizational character may create value not only by directly enhancing business performance and developing growth potential, but also by exerting an influence on the external environment. From the resource-based view, character nurtured and developed by an organization can be regarded as a kind of scarce and inimitable asset that can become the source of competitive advantages. Different types of organizational character will have different effects on organizational management and brand image. An effective combination of different dimensions of organizational character will have significant effects on organizational performance in a given situation.

It should be recognized that the study was carried out in a Chinese context. Compared with the organizational character dimensions previously defined by relevant literature in developed countries, Chinese enterprises' organizational character inherits oriental cultural traditions and retains its unique indigenous characteristics. In addition, Chinese organizational character is also inevitably affected by Western culture given the exchange and integration of China's economic and culture with the world. This is also the inevitable result of China's transition to modernization. Concretely, four dimensions including “boy scout,” “innovation,” “agreeableness,” and “conscientiousness” have strong cross-cultural consistency. This is a commonality of the explored dimensions in this study and in previous studies.

The difference is that “democracy” is discovered and explored as one of the most culturally unique dimensions of Chinese organizational character. One of the reasons may be that organizations in China often pay more attention to social relationships when compared to those in regions such as Europe and the United States. In these developed regions, organizational justice is universal in enterprises and to most of their employees. However, in China, along with the rapid development through the reform and opening up, many problems such as the imbalance of regional development, the large income gap between the rich and the poor, the insufficient protection of citizens' rights and interests, and the corruption of public power has raised and affected social justice. The fact that the promotion of social fairness and justice was repeatedly listed as a prior issue in the national strategy of the seventeenth and eighteenth national congress of the Communist Party of China indicates that democracy and justice have become serious realistic problems to which the party and the government should attach great importance. From a micro level, because of the coexistence of various ownership systems, distribution systems, and distribution forms as well as personnel systems, the distribution system for organizational development is unjust, unreasonable, and imperfect to a certain extent, and their employees' awareness of fairness and democracy is therefore becoming more and more intense. If the distribution in an organization is compared to a game, for Western researchers and managers, the problem is how to implement the operational and distributional rules fairly and effectively under the precondition of the universally recognized rules of the game; however, in China, a more realistic problem is to guarantee and increase the fairness of the game rules. It was reversely proven in our survey that many respondents from Chinese state-owned corporations thought that the “bureaucracy” was serious and general in their organizations. Another survey also found that about 60% of people believe that the public and administrative power is an important factor leading to the unfairness in Chinese society (Li, 2006). Therefore, it was not unexpected that we extracted the “democracy” dimension in a Chinese context, while it has not been emphasized in previous studies based on other cultural situations, when Chinese employees pay great attention to the feelings of democracy or bureaucracy, as well as openness and fairness.

Indeed, organizational character is the embodiment of the difference between two different social cultures. Since enterprises need to survive and develop in society, their behaviors should be influenced by external environment factors such as culture, policies, regulations, and strategies of the state. As far as the outside factors of organizations are concerned, a contingency model of organizational character evolution should be constructed from the perspective of environmental–organizational interaction development to further deepen the research about the formation mechanism of organizational character.

The impacting paths indicate that organizational character is positive for the advancement of organizational performance including business performance and growth potential. Concretely, character dimensions including enterprise, conscientiousness, innovation, and boy scout have significant positive effects on current business performance, while enterprise, conscientiousness, innovation, agreeableness, and democracy affect the long-term growth potential. These conclusions not only imply to us that enterprises could raise their performance through cultivating and utilizing organizational characters, but also advise them on how to cultivate different dimensions of organizational character and select and combine them to better realize their performance targets. Longer term, organizations can even form core competencies and gain sustainable competitive advantage through the impacting paths driven by organizational character. Particularly in a Chinese context full of complexity and uncertainty, organizations could increase the probabilities of survival and development when they face competitive pressure, environmental shocks, and transformation demand.

At present, the Chinese economy and society are undergoing tremendous transformation and change. Enterprises are facing a unique external environment. The importance of innovation to the survival and sustainable development of organizations has become increasingly prominent. Some enterprises (such as HUAWEI, Alibaba, and so on) have been continuously innovating while others are facing innovative difficulties. Could organizational character provide a new solution for those enterprises? How to explore and cultivate their organizational character, and apply it to advance organizational performance? The answers to these questions need further study.

## Conclusions

Based on the theory of organizational character, this study combined qualitative and quantitative methods to measure organizational characters and put forward an analytical framework with six dimensions in the Chinese context. Moreover, the impacting paths between organizational character and organizational performance were observed. By conducting an empirical study, we found that organizational character had significantly positive impacts on organizational performance including both the business performance and growth potential. It was revealed that along the paths, organizational character would turn into a necessary source of sustainable competitive advantage.

The study discussed how to nurture specific organizational characters to improve organizational performance in China. It may make the following possible contributions in theory and practice. First, although scholars have paid attention to organizational character as early as the 1990s, the research tends to move along at a slow pace on this theme. The framework of organizational character proposed in this paper fills the gap of prior studies in a systematic way and can be regarded as a breakthrough for promoting the research of organizational character. Furthermore, this study explored the relationships between organizational character and organizational performance and obtained some enlightening findings, which could provide a new perspective for scholars to enrich the source of competitive advantages. Last, but not the least, in practice, the results showed that top managers could intentionally develop some traits of organizational character to improve organizational performance. We also suggest that organizations in real life should form and insist on the cultivation of their characters for improving the business environment as well as creating sustainable values for themselves.

Furthermore, the organizations with unique and distinctive characters usually need to be supported by knowledge and technology to steadily improve their operational efficiency and enhance the inimitability of maintaining their core competitiveness. Thus, in the future, we will study the synergistic mechanisms of organizational character and knowledge capital, and further devote ourselves to exploring their impacts on the formation and promotion of competitive advantages. In turn, we hope to find a way to guide organizations to achieve “intellectual beauty,” which is a kind of mysterious oriental aesthetic complexly supported by knowledge-based characters. This section is not mandatory, but may be added if there are patents resulting from the work reported in this manuscript.

## Ethics statement

The data of this study were collected by questionnaire survey through a professional service institute. The content of the questionnaire was not involved with any ethical problem. All respondents were informed of the aims of this research and it was indicated that they approved this study when they filled out the questionnaire. For these reasons, the authors considered that we did not need to provide ethics approval and written informed consent.

## Author contributions

DY designed and performed the research. HX collected and processed the data and QB wrote the paper. All authors reviewed, edited, and approved the final manuscript.

### Conflict of interest statement

The authors declare that the research was conducted in the absence of any commercial or financial relationships that could be construed as a potential conflict of interest.
